# Combination of Osimertinib and Brigatinib in the Treatment of EGFR Triple-Mutated Lung Adenocarcinoma: A Case Report

**DOI:** 10.3390/curroncol32050270

**Published:** 2025-05-07

**Authors:** Daphnée Demers, Marie Florescu

**Affiliations:** 1 Faculty of Medicine, McGill University, Montreal, QC H3G 2M1, Canada; 2 Division of Hematology-Oncology, Centre Hospitalier de l’Université de Montréal (CHUM), Montreal, QC H2X 0C1, Canada

**Keywords:** NSCLC, EGFR, acquired resistance, tyrosine kinase inhibitors, osimertinib, brigatinib, toxicity

## Abstract

Osimertinib, a third-generation epidermal growth factor receptor (EGFR) tyrosine kinase inhibitor (TKI), is widely used in treating patients with EGFR-mutated non-small-cell lung cancers (NSCLCs), especially in cases with secondary resistance mutations. However, tertiary resistance mutations often arise, and there is currently no established standard of care for NSCLC harboring triple EGFR mutations. In recent years, brigatinib, an anaplastic lymphoma kinase (ALK) TKI, has shown effectiveness in treating EGFR triple-mutated NSCLC. Despite this, the combined use of osimertinib and brigatinib remains largely unstudied. This case report describes a 51-year-old woman with EGFR-mutated NSCLC who was initially treated with first- and second-generation EGFR TKIs, then switched to osimertinib upon development of an exon 20 T790M mutation. When an exon 20 C797S mutation emerged, the decision was made to add brigatinib to the osimertinib regimen. The combined treatment of osimertinib and brigatinib offers a promising new approach. Nonetheless, it is important to consider the potential risk of off-target toxicities.

## 1. Introduction

Lung cancer remains the most frequently diagnosed cancer in Canada and the leading cause of cancer-related deaths for both men and women [1]. Non-small-cell lung cancer (NSCLC), which accounts for 85% of lung cancer cases, is often characterized by driver alterations. Mutations in the epidermal growth factor receptor (EGFR) gene are among those recognized as potent therapeutic targets. Currently, these mutations are detected in 10–15% of NSCLC cases in Western populations and 40–60% in Asian populations [2].

EGFR tyrosine kinase inhibitors (TKIs) have improved progression-free survival in NSCLC patients [3,4]. However, treatment with first-generation (gefitinib, erlotinib) and second-generation (afatinib, dacomitinib) EGFR TKIs frequently engenders molecular mechanisms of resistance. The most relevant is the T790M mutation in exon 20 of the EGFR gene, observed in 60% of patients with acquired resistance. The replacement of threonine with methionine hinders the binding of EGFR TKIs to the ATP-binding site of EGFR. The indicated treatment in such patients is the third-generation EGFR TKI osimertinib, which demonstrated favorable results as a salvage therapy, owing to its ability to bind to the cysteine residue at position 797 of the mutated receptor [5,6]. Nonetheless, resistance to osimertinib inevitably emerges. Although a variety of tertiary mutations are known, the most prevalent is the C797S mutation found in exon 20, which accounts for 10–26% of cases. This mutation involves the substitution of cysteine with serine, which disrupts the covalent bond between osimertinib and the receptor [7]. Currently, there is no standard treatment for EGFR triple mutation (activating-mutation/T790M/C797S).

Interestingly, a focused drug screening of 30 kinase inhibitors revealed that brigatinib, a TKI clinically used in NSCLC patients with alterations in the anaplastic lymphoma kinase (ALK) gene, had an inhibitory effect against cells expressing triple-mutant EGFR. This is attributed to its ability to competitively bind to the ATP-binding pocket without causing steric interference to T790M or C797S. Brigatinib efficacy against triple-mutant EGFR was demonstrated through in vitro and in vivo experiments [8], and a retrospective cohort study revealed that EGFR T790M-cis-C797S patients treated with brigatinib-based therapy experienced superior progression-free survival compared to those on alternative targeted treatments [9].

We present the case of a patient diagnosed with a lung adenocarcinoma harboring an EGFR del19/T790M/C797S triple mutation, who underwent treatment involving a combination of brigatinib and osimertinib.

## 2. Case Description

**Figure 1:**
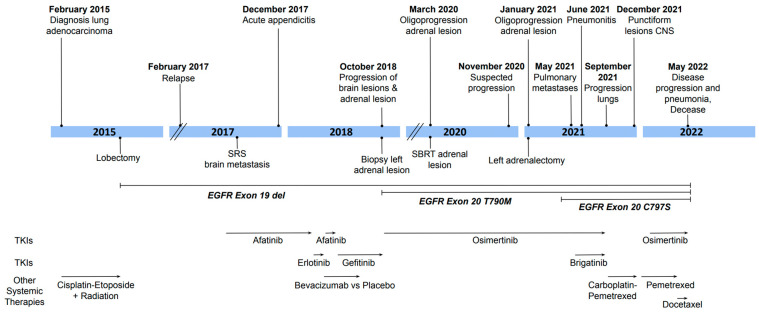
Timeline of patient’s diagnosis and treatments.

A 51-year-old woman, former light smoker (10 pack years), presented with persistent cough and dyspnea in February 2015. She was diagnosed with locally advanced lung adenocarcinoma (stage IIIB: T3 N2 M0). She initially received four cycles of neo-adjuvant cisplatin and etoposide, as well as radiation therapy. She then underwent a right lower lobectomy with a wedge resection at the level of the right middle lobe. She remained without evidence of disease until February 2017. At that time, she complained of occasional headaches but had no respiratory or systemic symptoms. Lesions were found in the right lung, left adrenal gland, and right insula. A biopsy performed on the left adrenal mass confirmed the diagnosis of adenocarcinoma recurrence, and PCR analysis conducted on the biopsy specimen indicated the presence of an EGFR exon 19 deletion. The patient’s clinical course from the time of diagnosis is illustrated in Figure 1.

In July 2017, EGFR TKI therapy with afatinib was initiated. The patient developed dermatologic side effects, including an erythematous, acneiform rash and mouth ulcers. A brief trial of erlotinib was attempted; however, she experienced an allergic reaction. In August 2017, she underwent robotic stereotactic radiosurgery (SRS) with 20 Gy in one fraction for the right insular metastasis. This approach successfully achieved local control, as demonstrated by a follow-up positron emission tomography (PET) scan in September showing resolution of the lesion. In March 2018, radionecrosis at the level of the central nervous system emerged, leading to the patient’s enrollment in a protocol comparing the efficacy of bevacizumab vs. placebo. She received four cycles and responded favorably. During this time, she was also treated with gefitinib.

In October 2018, 15 months after initiating treatment with first- and second-generation EGFR TKIs, there was significant growth of the left adrenal lesion as well as new brain lesions. A biopsy of the left adrenal lesion unveiled the EGFR exon 20 T790M secondary mutation, identified using next-generation sequencing (NGS). Thus, the patient’s treatment was changed to osimertinib. In September 2020, she achieved remission. However, in January 2021, oligoprogression of the adrenal lesion led to a left adrenalectomy. In May 2021, the patient experienced worsening dyspnea, accompanied by progression of the cancerous pulmonary micronodules. Pathological analysis of the previously excised adrenal metastatic lesion using NGS revealed the presence of an additional EGFR mutation, this time in exon 20 C797S.

As a result, starting from June 2021, brigatinib (90 mg/d) was introduced in combination with osimertinib. Osimertinib was maintained due to its ability to penetrate the central nervous system, including cerebrospinal fluid, and the patient’s existing resistance to other treatment lines. In the initial 48 h after beginning this treatment, the patient experienced significant dyspnea. After two days of using a salmeterol and fluticasone inhaler, the symptoms resolved without the need for further intervention. Therefore, the dosage of brigatinib was increased to the recommended 180 mg per day. While this did not lead to dyspnea, there seemed to be an unavoidable pattern of toxicity, as the patient began experiencing erythromelalgia with skin fissures, desquamation, transaminitis, myositis, and paresthesia. She also exhibited paronychia. When assessed in August, she had no respiratory complaints, but as a response to the observed toxicity, the dose of brigatinib was gradually reduced.

**Figure 2:**
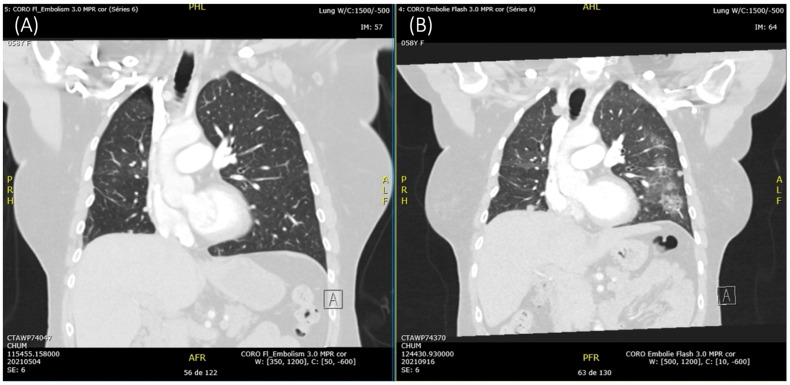
Angioscan of the thorax from (**A**) May 2021, approximately a month prior to the initiation of brigatinib, and (**B**) September 2021.

In September 2021, three months after initiation of dual TKIs, the patient was admitted to the oncology floor due to dyspnea. She underwent an angioscan, which showed that not only had the disease progressed in the lungs, but she had developed pneumopathy (Figure 2). An infectious cause was excluded via bronchoalveolar lavage, and the diagnosis of pneumonitis resulting from the use of TKIs was established. Both osimertinib and brigatinib were discontinued. She received supportive therapy as well as dexamethasone, in preparation for her chemotherapy treatment. Within 6 weeks, her pneumonitis had resolved.

Moreover, the patient’s C-reactive protein level was moderately elevated at 29 mg/L, and a PET scan suggested that the inflammation might have extended beyond the lungs, as diffuse activity was observed in approximately one-third of the esophagus. There was also evidence of hepatotoxicity. On 24 September, her liver injury markers were as follows: alanine transaminase (ALT) 312 U/L, aspartate transaminase (AST) 214 U/L, alkaline phosphatase 223 U/L, and lactate dehydrogenase 379 U/L. They normalized shortly after the discontinuation of dual TKIs.

The patient then began treatment with carboplatin pemetrexed. An MRI conducted 3 months later showed meningeal carcinomatosis with multiple micronodules. Consequently, osimertinib was resumed, at a dose of 40 mg daily instead of the usual 80 mg, to avoid toxicities. She did not experience any. By March, all brain lesions had disappeared, yet the disease had progressed in the lungs. Treatment was modified to docetaxel and osimertinib. In May 2022, the patient was admitted to the hospital, where she passed away from pulmonary deterioration.

## 3. Discussion

The emergence of EGFR triple mutations is significant, as they represent a complex resistance mechanism that poses a substantial challenge to treatment. Currently, there is no effective treatment for EGFR triple-mutated NSCLC. Therefore, TKI combination therapies may provide potential novel treatment options for patients. A prior case report revealed that osimertinib, bevacizumab, and brigatinib combination treatment relieved tumor growth and respiratory symptoms in a patient with adenocarcinoma harboring an EGFR L858R/T790M/C797S triple mutation [10]. The present case adds to the limited body of literature exploring the combination of osimertinib and brigatinib in EGFR-mutated NSCLC. Indeed, while both agents have demonstrated efficacy individually, their combined use remains largely unstudied. It provides insights into the potential challenges of dual-TKI therapy, including off-target side effects.

Currently, brigatinib is approved as a first-line treatment for patients with ALK-positive locally advanced or metastatic NSCLC [11]. The existing literature suggests that adverse events associated with brigatinib are typically manageable, the most common ones being increased creatine phosphokinase, diarrhea, and nausea [12], though there are limited data on the safety of brigatinib monotherapy in EGFR-mutated NSCLC.

On the other hand, the EGFR signaling pathway is important in maintaining skin functions, such as wound healing, inflammation regulation, and capillary constriction. Hence, dermatological toxicities are the most common adverse reactions caused by EGFR inhibitors. Their occurrence is nonetheless reduced in osimertinib, owing to its enhanced activity against tumor EGFR carrying the mutations del19, L858R, and T790M, compared to wild-type EGFR [13]. In the FLAURA phase 3 trial, 58% of patients treated with osimertinib developed a rash or acne, as opposed to 78% with standard EGFR TKI [14]. Conversely, brigatinib is associated with a lower frequency of skin-related adverse events, with dermatitis acneiform present in 7% of patients and rash in 10% [15]. Our patient received treatment with osimertinib for over two years without experiencing mucocutaneous side effects. Yet, the combination of osimertinib with brigatinib likely precipitated such reactions, as various skin toxicities emerged upon the addition of brigatinib. She was most bothered by the erythromelalgia with skin fissures, paresthesia, and paronychia. She received treatment with a topical antibiotic, fusidic acid, and oral amoxicillin with clavulanic acid. As per recommendations, a topical corticosteroid was also prescribed, in her case clobetasol propionate [13]. Nevertheless, the cutaneous toxicities persisted until osimertinib and brigatinib were discontinued. They did not recur when osimertinib was later reintroduced.

While uncommon, severe inflammation of the lungs is a documented potential side effect of brigatinib. The ALTA trial reported pulmonary toxicity rates of 3.7% in the 90 mg group and 9.1% in the 180 mg group. Within the first 9 days of initiating brigatinib therapy, pneumonitis developed in 6.4% of patients, with a median onset of 2 days [16]. The occurrence of pneumonitis beyond the initial few weeks, as observed in our patient, is uncommon. Moreover, the first reported case of dual-TKI pulmonary toxicities emerged when a patient carrying the L858R/T790M/cisC797S mutation experienced early-onset pulmonary events with bilateral interstitial infiltrates after being treated with concurrent brigatinib and afatinib [17].

Grade 3 elevation in serum hepatic enzymes as a result of brigatinib treatment, as seen in our patient, occurs in only 1% to 3% of instances [18]. The incidence of this in the case of osimertinib is less than 1% [19]. Our patient did not encounter notable elevation in hepatic enzymes with osimertinib or any of the other TKIs she had previously received. She was not taking any other medication that might have impacted her liver function. While direct interaction between brigatinib and osimertinib has not been widely reported, their shared metabolism via the hepatic enzyme CYP3A4 suggests that concurrent use could potentially alter drug levels, and the possibility of hepatic interaction cannot be excluded [20]. The limitation of the present report is that we did not measure the actual plasma concentrations of brigatinib and osimertinib, either when administered alone or concurrently.

Robotic SRS was performed in August 2017 to treat the patient’s insular brain metastasis, one month after initiating afatinib. CNS radionecrosis was identified the following March. The extent to which concurrent TKI therapy contributed to radionecrosis in addition to radiotherapy remains unclear. Preclinical studies suggest that TKIs may potentiate the effects of radiation, though their impact on normal tissue remains unclear. A systematic review highlighted that combining cranial radiotherapy with TKIs in NSCLC patients can lead to increased neurotoxicity, including radionecrosis [21].

Herein, we found that the combination of brigatinib and osimertinib was not effective for NSCLC with a del19/T790M/C797S triple mutation, as it provided no clinical benefit and engendered grade 2 and 3 toxicities that led to treatment discontinuation. Unanticipatedly, in a patient who tolerated osimertinib very well, the addition of brigatinib generated off-target side effects, such as skin-related toxicities and hepatic injury. It is unclear whether the pneumonitis was caused by either TKI or the combination of the two. The patient was reintroduced to osimertinib later in the course, and once again, it was well tolerated. Thus, the increased risk of off-target toxicities, notably dermatologic, pulmonary, and hepatic, should be kept in mind when using osimertinib and brigatinib concurrently. Additionally, as mentioned in the literature, while brigatinib is effective against osimertinib-resistant EGFR mutants, tumor relapse, as observed in this case, suggests that additional resistance mutations might emerge [22].

## 4. Conclusions

The existing literature indicates efficacy of brigatinib therapy in triple-mutated NSCLC, as a monotherapy and in combination with various agents. Both brigatinib and osimertinib are approved and accessible in clinical practice and have favorable side effect profiles. Their oral administration also contributes to patient adherence and acceptance. However, there is a lack of data regarding concentrations, safety, and effectiveness when used together, specifically in patients with triple-mutated EGFR NSCLC. Further reports are needed to establish the safety and efficacy of brigatinib and osimertinib combination therapy.

## Data Availability

The original contributions presented in this study are included in the article. Further inquiries can be directed to the corresponding author.
